# Manufacturing of Carbon Fibers/Polyphenylene Sulfide Composites via Induction-Heating Molding: Morphology, Mechanical Properties, and Flammability

**DOI:** 10.3390/polym14214587

**Published:** 2022-10-28

**Authors:** Chang-Soo Kang, Hyun-Kyu Shin, Yong-Sik Chung, Min-Kang Seo, Bo-Kyung Choi

**Affiliations:** 1 Korea Carbon Industry Promotion Agency, Jeonju 54853, Korea; 2 Department of Organic Materials & Fiber Engineering, Jeonbuk National University, Jeonju 54896, Korea

**Keywords:** thermoplastic composites, induction-heating molding, microstructure, mechanical properties, flammability

## Abstract

Conventional thermosetting composites exhibit advantageous mechanical properties owing to the use of an autoclave; however, their wide usage is limited by high production costs and long molding times. In contrast, the fabrication of thermoplastic composites involves out-of-autoclave processes that use press equipment. In particular, induction-heating molding facilitates a quicker thermal cycle, reduced processing time, and improved durability of the thermoplastic polymers; thus, the process cost and production time can be reduced. In this study, carbon fiber/polyphenylene sulfide thermoplastic composites were manufactured using induction-heating molding, and the relationships among the process, structure, and mechanical properties were investigated. The composites were characterized using optical and scanning electron microscopy, an ultrasonic C-scan, and X-ray computed tomography. In addition, the composites were subjected to flammability tests. This study provides novel insights into the optimization of thermoplastic composite manufacturing and thermoset composite curing processes.

## 1. Introduction

Carbon fiber-reinforced polymer (CFRP) composites are widely used in various industries, such as transportation, construction, electronics, aerospace, automotive, and marine, because of their excellent stiffness and strength at low bulk densities [1,2,3]. In particular, the aerospace industry is a global market that has a high demand for high-performance composites and thermoset resins, such as epoxy [4,5]. Thermoset resins offer advantages such as excellent physical properties and facile impregnation owing to their low viscosity. However, they require a relatively long curing time, and their use is limited by difficulties in storing and handling the raw materials. Consequently, there is increasing interest in exploring thermoplastic resins as alternatives to overcome these limitations [6,7,8,9,10]. 

In the past decades, research on CF-reinforced thermoplastic composites has primarily focused on polymers, such as polyether ether ketone (PEEK), polyamide 6 (PA6), polypropylene (PP), and polyphenylene sulfide (PPS) [11,12,13]. Among them, as a semi-crystalline specialty thermoplastic, PPS is well known because of its high heat resistance and flame retardancy, outstanding chemical resistance, dimensional stability, and low toxicity. In addition, PPS has a high degree of crystallinity (~60%) and maintains its mechanical properties well when exposed to fire [14,15]. 

Although conventional composites exhibit superior mechanical properties owing to the use of high temperatures and pressures in autoclaves, their use is limited by high production costs and long molding times. In contrast, an out-of-autoclave (OoA) process such as thermoforming, which uses a press, can be performed at lower processing costs with higher production efficiency [16,17,18].

Hot presses are commonly used in the manufacturing of CFRP composites, and can efficiently attain the high temperatures and pressures required to ensure the complete impregnation of thermoplastic composites [19]. However, their impregnation capabilities deteriorate because of the difficulty in achieving complete resin impregnation during molding. This leads to fiber dislocation and the generation of internal voids, causing the physical and mechanical properties to deteriorate [20]. In addition, press heaters exhibit limited heating rates, usually below 5 °C/min, and have a high processing time [21]. Chen et al. [22] studied the effect of automated fiber placement (AFP) processing parameters on the mechanical properties of CF/PPS composites from the perspective of void content and crystallinity. They found that the interlaminar void content, rather than the crystallinity, dominated the mechanical properties of the composites. Until recently, the use of thermoplastic composites has been limited owing to impregnation difficulties (pore and void control) and high processing temperature requirements. To overcome these issues, various processes have been developed to improve the impregnation capability of reinforcing fibers using high-performance thermoplastic resins. Ramakrishnan et al. [23] manufactured lightweight commingled flax/PP thermoplastic biocomposites using fast induction-heating compression molding. They also investigated the effects of different pressure and temperature cycles on the consolidation quality of the flax/PP biocomposites, and proposed an optimum processing cycle to reduce the manufacturing time without significantly affecting the mechanical properties of the biocomposites.

Induction-heating molding affords a quicker thermal cycle, reduced processing time, and improved durability of thermoplastic polymers. Moreover, a significant reduction in electricity consumption is achieved. Therefore, induction-heating molding is an efficient approach to reduce the manufacturing costs of composites [24]. Thus, it is determined that thermoplastic composite materials, which are intended to replace materials such as metal and steel, can make contributions to the aerospace industry in terms of cost in the long term, with their properties such as recyclability and storability in room conditions.

In this study, thermoplastic composites were manufactured via an induction-heating molding process using CF/PPS prepreg unidirectional (UD) tape. The process involved high heating and cooling rates, leading to shorter compression cycles. Mechanical tests were conducted to determine the influence of processing conditions and hence, better understand the process-microstructure-property relationship.

## 2. Experimental

### 2.1. Materials

The thermoplastic prepreg used in this study was a CF/PPS UD tape (CETEX^®^ TC1100 PPS, Toray Advanced Composites, California, USA) with a fiber areal weight of 221 g/m^2^. The initial weight fraction of the prepreg matrix was 34%, and consisted of a PPS matrix and CF (AS4A fiber with a volume fraction of 59%). The properties of the tape are listed in Table 1. 

### 2.2. Composite Manufacturing and Sample Preparation

**Figure 1:**
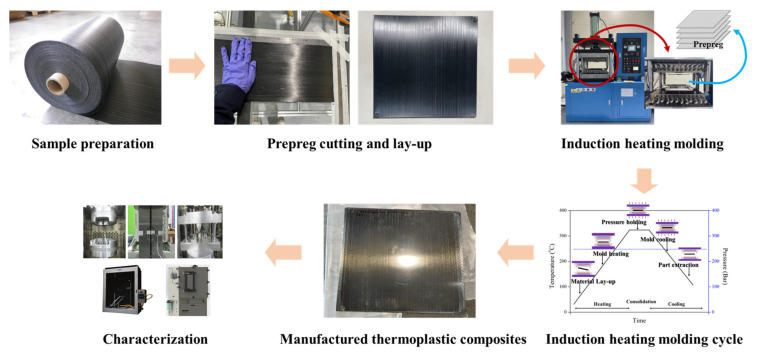
Schematic of the CF/PPS composites manufacturing process using induction-heating molding.

**Figure 2:**
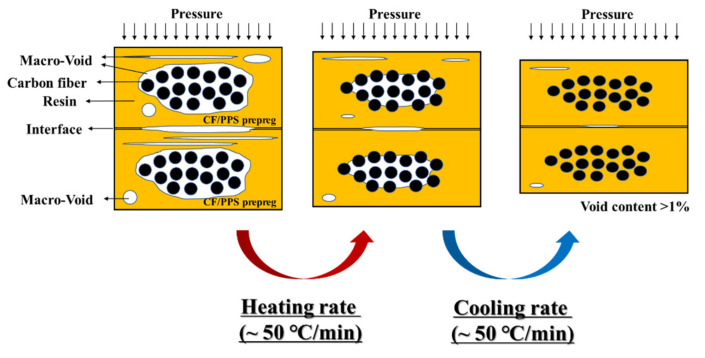
Schematic of the CF/PPS prepreg consolidation process.

The stacking sequence for the laminates was 0°, and the final dimensions of the thermoplastic sheets were 300 × 300 × 1~3 mm (length × width × thickness). The thermal cycle consisted of a heating phase at 40 °C/min until 250, 280, and 310 °C. There was an isotherm stage at this temperature during 5 to 30 min under a pressure of 9 to 50 bar, and the cooling stage was at 20 °C/min. The laminates were laid up and each ply was manually fixed using a small welding point. A schematic of the CF/PPS composites manufacturing process via induction-heating molding is shown in Figure 1. After the lay-up, the unconsolidated laminate composites consisted of partially impregnated plies. The macro-voids were produced during the cooling step because of the mold contraction. The loss of the mold curvature during the cooling lead to the composite deconsolidation before the matrix solidification, which was observed in the matrix zones. Each ply consisted of the aligned fiber tows, micro-voids between the individual fiber tows, and macro-voids in the resin regions located around the tows and between the plies. Once the pressure was applied, the air escaped through the dry tow cores, which decreased the macro-void content and compacted the fiber bed, and the tows remained partially dry. Once the temperature was raised, the resin infiltrated the dry tow areas, which resulted in a void-free microstructure. (For the crimped reinforcements, some flow might occur along the fiber direction.) Each ply was reduced to a uniform final cured ply thickness with the no voids. A schematic of the CF/PPS prepreg consolidation process is shown in Figure 2. 

**Figure 3:**
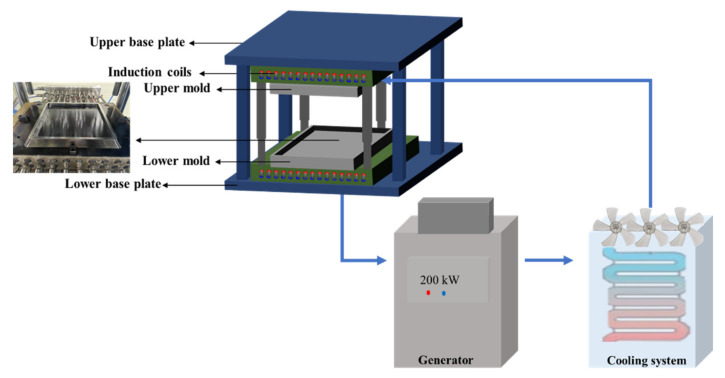
Schematic of the induction-heating molding system.

The CF/PPS UD tape was molded into laminates by applying a heating and pressure cycle to melt the PPS resin, followed by a cooling cycle for PPS crystallization and consolidation of the laminates. Figure 3 shows the induction-heating molding system (3iTech^®^, Roctool, Le Bourget du Lac, France) employed, which consists of a vertical press (capacity: 100 kN), a 200 kW medium-frequency induction generator, a water-cooling unit capable of supplying cold water up to 100 L/min, and a 300 × 300 mm maximum molding area.

### 2.3. Characterization

#### 2.3.1. Microstructural Properties

An optical digital image analysis system equipped with a digital camera (KCX-20B, Korea Lab Tech., Seongnam, Korea) was used to observe the morphology of the composites. The CF/PPS composites were subjected to an ultrasonic C-scan (SDI 5380, CBOL Corporation, CA, USA) to detect the presence of defects (pores or voids). Micro-focus X-ray computed tomography (CT, SMX-225CTS, Shimadzu, Kyoto, Japan) was employed for nondestructive visualization to estimate the void content of the composites. The micro-CT instrument was equipped with a flat laminate detector (512 × 512 pixels) and a micro-focus X-ray source (170 kV and 190 μA).

#### 2.3.2. Thermal Properties

The degree of crystallinity (*x*) of the composites was determined using differential scanning calorimetry (DSC, Q20, TA Instruments, New Castle, DE, USA). The samples (5–10 mg) were heated from 30 to 400 °C at a rate of 10 °C/min in a nitrogen atmosphere, and *x* was calculated using Equation (1):

(1) $$x\;\left(\%\right)=\frac{\Delta H_{m}-\Delta H_{c}}{\Delta H_{f}\left(1-\alpha \right)}\;$$

where Δ*H_m_* is the enthalpy measured from the melting peak, Δ*H_c_* is the enthalpy of cold crystallization, Δ*H_f_* is the enthalpy of melting of a theoretical 100% crystalline polymer (150.4 J/g) [25], and α is the CF mass fraction in the composites. At least three samples were tested for each composite, and the average value was considered as the final degree of crystallinity.

#### 2.3.3. Mechanical Properties

Tensile and interlaminar shear test samples were prepared to evaluate the mechanical properties of the composites. The samples were cut from the laminate and tabbed with the same composite laminate coupons using high-strength epoxy glue. All tests were performed using a universal testing machine (Instron 5982, Instron, OR, USA) equipped with a 100 kN load cell. 

The tensile tests of at least five samples for each composite were conducted according to the ASTM D3039 standard at a crosshead speed of 2 mm/min. The tensile strength was calculated using Equation (2):

(2) $$\;F^{tu}=\frac{P_{max}}{A}\;$$

where *F^tu^* is the ultimate tensile strength (MPa), *P_max_* is the maximum load before failure (N), and A is the average cross-sectional area (mm^2^).

The interlaminar shear strength (ILSS) test is commonly used to characterize the interlaminar failure resistance of composites. The method involves loading a short beam under three-point bending such that interlaminar shear failure is induced. The ILSS tests of the composites were performed according to the ASTM D2344 [26] standard. The span-to-thickness ratio of the specimen was 2:1, and the dimension of the ILSS test specimen were 18 mm × 6 mm × 3 mm. A minimum of five samples from each composite were tested at a crosshead speed of 1 mm/min. *F^sbs^* was calculated from the force-displacement curves using Equation (3):

(3) $$F^{sbs}=0.75\times \frac{P_{m}}{b\times h}\;$$

where *F^sbs^* is the short-beam strength (MPa), *P_m_* is the maximum flexural load (N), *b* is the sample width (mm), and *h* is the sample thickness (mm).

#### 2.3.4. Flammability Properties

Flammability tests were performed according to the ISO 5660-1 [27] standard using a cone calorimeter (CC 2006, FESTEC Co. Ltd., Seoul, Korea) at a heat flux of 25 kW/m^2^ and for a duration of 10 min. The samples (100 mm × 10 mm × 1 mm) were wrapped on the side and bottom with aluminum foil and placed horizontally on the sample holder.

**Figure 4:**
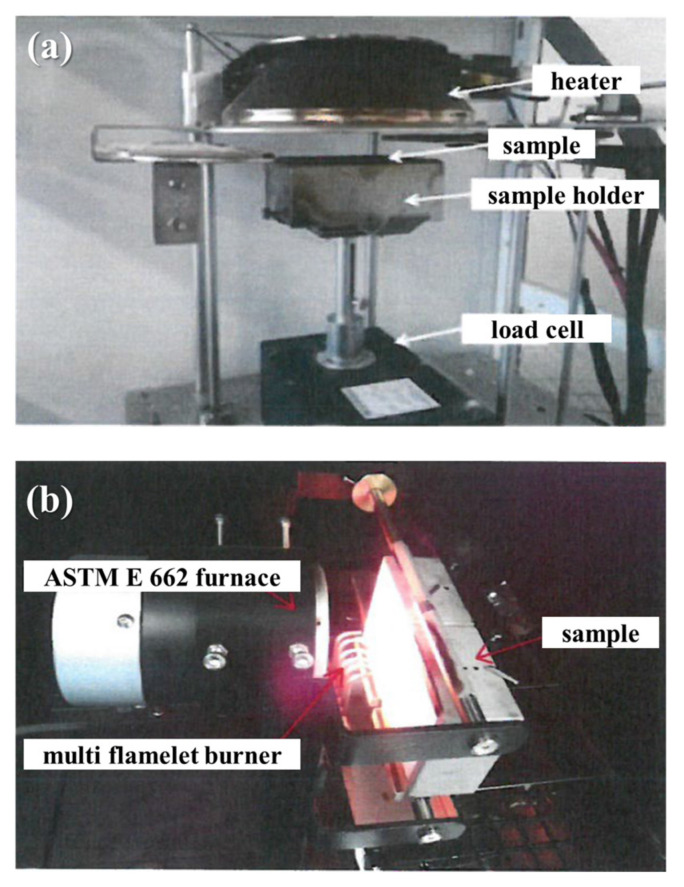
Photographs of (**a**) cone calorimeter and (**b**) smoke density tester.

The optical density of the smoke was assessed according to the ASTM E662 [28] standard using a smoke density tester (FT-SB-501, FESTEC Co. Ltd., Seoul, Korea). Each sample (75 mm × 75 mm × 1 mm) was covered with aluminum foil and exposed vertically to a heat flux of 25 kW/m^2^ using a flame for a maximum duration of 20 min. A light beam was then passed through the chamber. The smoke density was calculated by measuring the obscuration of the beam by the smoke using a photosensor. VOF4 refers to the total optical density measured in the first 4 min of sample exposure to heat flux and was calculated using Equation (4):

(4) $$\mathrm{VOF}4=D_{s\left(1\right)}+D_{s\left(2\right)}+D_{s\left(3\right)}+\left(\frac{D_{S\left(4\right)}}{2}\right)\;$$

where D_s(1)_, D_s(2)_, D_s(3)_, and D_s(4)_ are the specific optical densities recorded at 1, 2, 3, and 4 min, respectively. Photographs of the cone calorimeter and the smoke density tester are shown in Figure 4. 

The toxic gases were analyzed according to the BS 6853 [29] standard (Annex B) using the smoke density tester coupled with a Fourier transform infrared spectrometer (I1801-E, Midac, MI, USA). Tests were performed with an irradiance level of 25 kW/m^2^ in the flame mode for a duration of 10 min. The gases were sampled from 650 to 4500 cm^−1^. The optical density of the smoke (D_S_) was calculated according to Equation (5):

(5) $$D_{S}=G\times \left(\log_{10}\frac{100}{T}\right)\;$$

where G = (V/A) × L, V is the volume of the closed chamber (m^3^), A is the exposed area of the sample (m^2^), L is the length of the light path through the smoke (i.e., 132), and T is the light transmittance (%). 

The toxicity index (R) was calculated using Equations (6) and (7):

(6) $$r_{x}=c_{x}/f_{x}\;$$

(7) R=∑r 

where r_x_ is the individual index for the x chemical species generated by the combustion of the sample, c_x_ is the emission of the x species in appropriate units, and f_x_ is the reference value for the x species, as listed in Table 2.

## 3. Results and Discussion

### 3.1. Microstructural Properties

**Figure 5:**
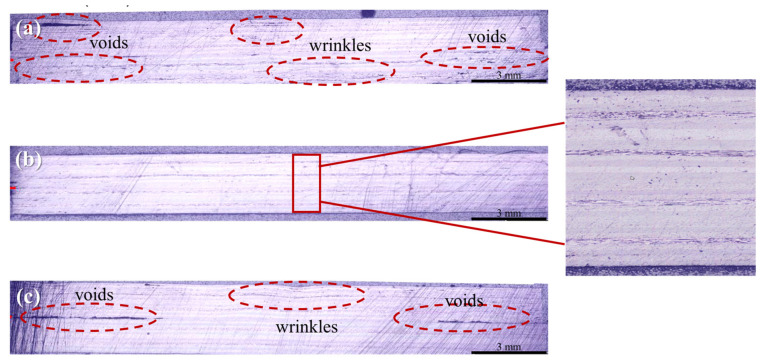
Cross-sectional optical micrographs of the CF/PPS composites; samples obtained at processing temperatures of (**a**) 250 °C, (**b**) 280 °C, and (**c**) 310 °C via induction technology.

Figure 5 shows cross-sectional optical micrographs of the CF/PPS composites. Some voids and wrinkles are observed in the composite samples (Figure 5a), indicating that the inner and outer plies are defective because of their proximity to the press plates, where the temperature and pressure are the lowest. The use of a low processing temperature and pressure (250 °C and 20 MPa, respectively) leads to intralaminar voids with a void content over the 20% threshold. While the use of low processing pressures hinders the complete removal of entrapped air from the prepreg raw material, the application of excess pressure may cause other undesirable effects, such as wrinkles or fiber distortion [30]. In addition, the use of temperatures over 310 °C causes significant matrix degradation involving multiple defects, such as large volumetric voids in interlaminar and intralaminar regions and void connections that span over different plies [30]. The results of the application of processing temperatures of 280 °C may lead to identical levels of consolidation of the CF/PPS composites without voids and wrinkles.

**Figure 6:**
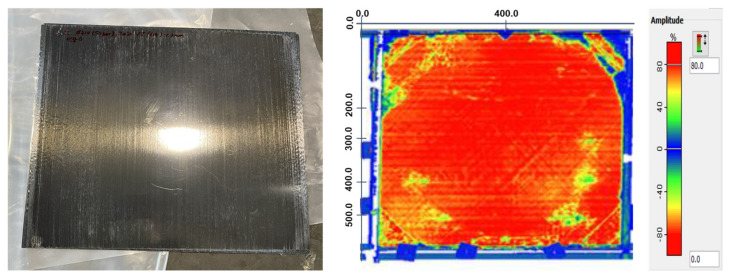
Photograph and C-scan image of a CF/PPS composite (processing temperatures of 280 °C).

The peeling distributions are determined via the depths of the laminates, and the presence or absence of defects are observed via the color difference, which is shown in Figure 6. The ultrasonic pulses were attenuated because of the irregularities inside the composites, such as voids and delaminated layers. The samples with unfavorable manufacturing conditions exhibited attenuated areas, which indicated that the entrapped air was not removed and the level of consolidation in the composites was not adequate for industrial applications. The C-scans indicated that most of the areas exhibited an attenuation level beyond 70%, but other areas of the plates were not consolidated, with spots observed near the edges attenuated less than 40%. In the C-scan analysis, the laminates with amplitude levels beyond 70% (volume echo; red color) were homogeneous and defect-free. In addition, an area amplitude of less than 60% (foreground echo; yellow color) and less than 40% (background echo; green color) were irregularities. The results showed that the application of the processing temperature of 280 °C may lead to identical levels of consolidation in the CF/PPS composites. This suggests that the low consolidation temperatures near the melting point of PPS obtained adequate levels of consolidation in the induction-heating molding. This provided a higher and quicker processing temperature for the suitable attenuation levels.

**Figure 7:**
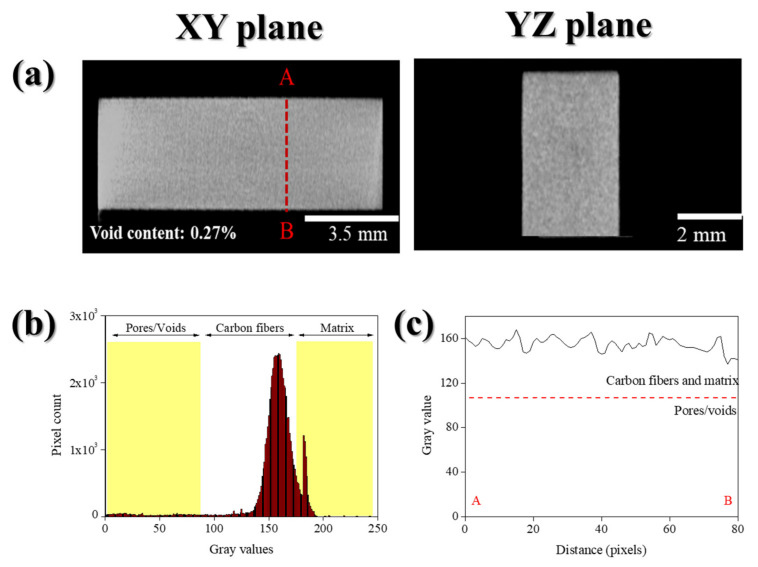
Tomographic slices of CF/PPS composites (processing temperatures of 280 °C): (**a**) X-ray computed tomography image, (**b**) histogram, and (**c**) line-profile.

Figure 7a shows the cross-sectional CT images of the CF/PPS composites. While pores or voids were rarely observed, X-ray tomography analysis identified the types of pores. The observed pore were cracks in the plies and transverse cracks in the composite samples, which exhibited a high void rate. Three levels were identified in the histograms of the X-ray CT images (Figure 7b): the left peak was the pore and void area; the middle peak was the CF region, and the right peak was the matrix area. The phase separation between the voids (the pores inside the composite and the background) and the carbon fiber-reinforced polymeric matrix were well defined. A line profile evaluated the line-to-line variation in the XY plane for the grayscale values in the analyzed sample (Figure 7c). The line probe determined the correlation between the grayscale intensity and the material composition, and between the cursor point position on the sample slice and the grayscale associated with the threshold values for each of the sample components. The voxels with a grayscale value less than 100 were classified as the pores, and those with a value higher than 100 were classified as the fibers and matrix [31,32].

### 3.2. Thermal Properties

**Figure 8:**
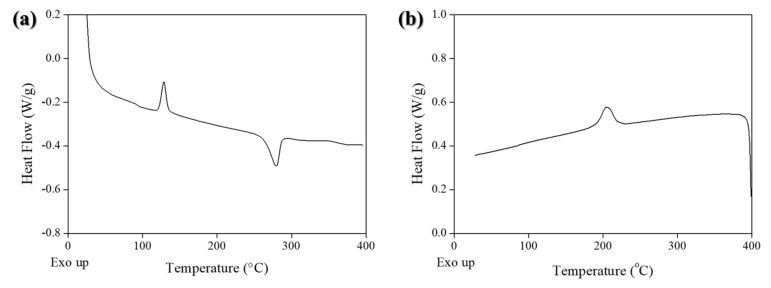
DSC curves for the CF/PPS composites (processing temperatures of 280 °C): (**a**) 1st heating and (**b**) 1st cooling cycles.

The DSC curves of the CF/PPS composites are shown in Figure 8, and Table 3 exhibits the average degrees of crystallinity of the composites manufactured at different temperatures. Crystallinity correlated to the cooling rate during the composite processing and the final mechanical properties of the laminates [19,33]. Saenz-Castillo et al. [19] reported the vacuum bag and hot press procedure in an oven with a low cooling rate of approximately 2 °C/min, and the laser-assisted in situ consolidation was characterized via ultra-fast cooling of up to 10,000 °C/min. Gao et al. [33] reported on the influence of the cooling rate on the final CF/PEEK crystallinity and its mechanical properties. The glass transition temperature of the PPS resin was 122.16 °C, which was higher than the value from the supplier (90 °C). The final melting temperature of PPS was 280.11 °C. The Δ*H_m_*, calculated from the area under the melting curve was 12.59 J/g, which corresponded to a degree of crystallinity of 25.3% via Equation (1) and was similar to the crystallinity value of neat PPS (25.88%). Thus, thermal treatment induced a change in the crystallinity because of the process temperature being higher than the melting temperature of the polymer [33].

### 3.3. Mechanical Properties

#### Tensile Strength and ILSS

**Figure 9:**
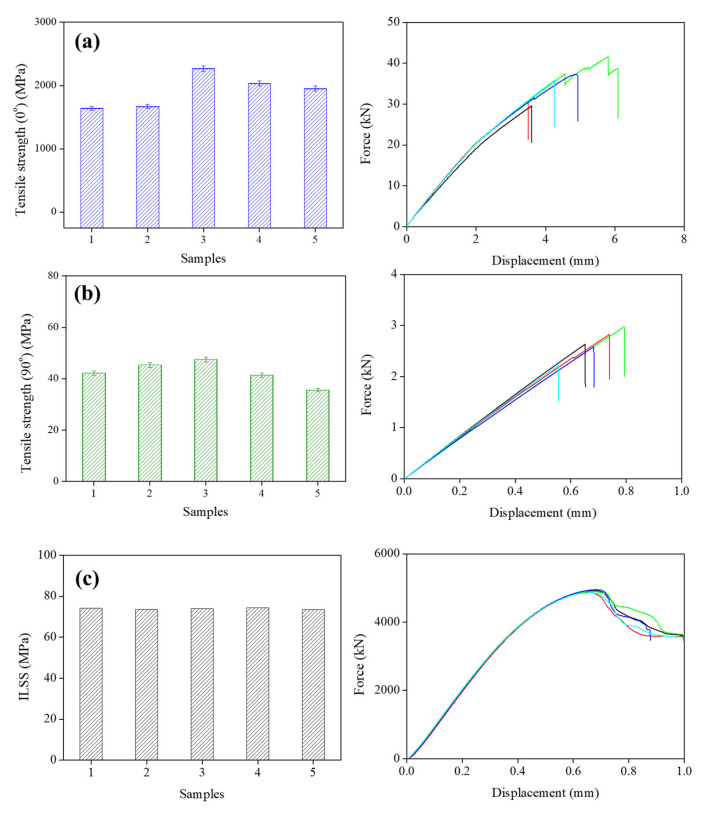
(**a**) Tensile strength (0°), (**b**) tensile strength (90°), and (**c**) ILSS of the CF/PPS composites (processing temperatures of 280 °C).

As shown in Figure 9a,b, the tensile strength of the CF/PPS composites is 1912.57 ± 262.58 MPa. The tensile strength of the pure PPS was 45.5 MPa, and the addition of CF increased this value to 91 MPa [34]. This proves that the CF/PPS composites were produced with a low void content and no delamination via the induction-heating molding process. Figure 9c shows the ILSS and force-displacement curves of the CF/PPS composites. The ILSS of the composite was 73.92 ± 0.41 MPa, and the main failure mode during the ILSS tests was the interlaminar shear between the CF layers. Chen et al. [22] reported the ILSS values of CF/PPS composites prepared via AFP. The reported average value (48 MPa) was lower than the induction-heating molding ILSS in this study (73.92 MPa). This difference resulted from the high crystallinity (>25%) of the material [22]. The ILSS values of the samples prepared in this study were higher than those prepared with/without an autoclave (65.9 and 49.2 MPa, respectively). The samples treated by the induction process in this study exhibited higher load values than the autoclave-treated samples because of their lower void contents and low extent of matrix degradation. The void contents in the autoclave-treated samples was approximately 3%, whereas that in the induction-heating molding samples was less than 1%, which was acceptable for the aerospace industry.

### 3.4. Flammability Properties

#### 3.4.1. Fire

The heat release rate (HRR) measured by a cone calorimeter is a very important parameter because it expresses the intensity of fire [35]. The HRR was measured according to the basic principle that 13.1 MJ of heat is generated when 1 kg of oxygen is consumed, and is defined as the quantity of heat released per unit area of a material when the material is subjected to a fire [35]. Parameters such as the time to ignition (TTI), peak heat release rate (PHRR), and total heat release (THR) were measured, and the maximum average rate of heat emission (MARHE) was calculated; the results are listed in Table 4. The TTI characterizes the time required for ignition when a material is exposed to a constant heat flux in an oxygen-controlled environment. The TTI reflects how quickly the surface of a material reaches the pyrolysis temperature and flammable volatile gases required to sustain the flame are produced over the entire sample surface. Hence, a higher TTI is more favorable and denotes less flammability. However, the TTI of the CF/PPS composites was recorded as zero, suggesting that they are non-ignitable materials, whereas the highest TTI for CF-reinforced bisphenol F and aniline-based benzoxazine [36] was 78 s. The CFRP and isotactic PP [37] exhibited the highest PHRR and MARHE values of 1738 kW/m^2^ and 94.6 kW/m^2^, respectively. For CFRP, PP, and polyamide 610 (PA610) [38], the same amount of heat was released in lesser time, which could lead to faster fire development.

#### 3.4.2. Smoke Density

**Figure 10:**
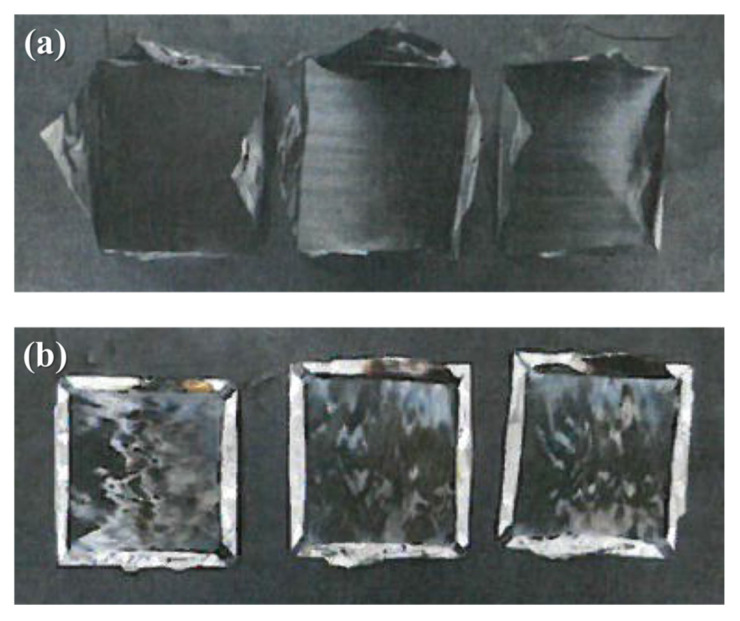
CF/PPS composites (processing temperatures of 280 °C) (**a**) before and (**b**) after smoke density tests.

The density of smoke at 10 min (D_s(10)_), maximum specific optical density (D_m_), and cumulative value of the specific optical density of smoke in the first 4 min (VOF4) are presented in Table 5. The D_s_ of the CF/PPS composites shows an almost linear increase after approximately 60 s. The CF/PPS composites emit a small amount of smoke with a lower D_s(10)_ than CFRP and PA610 at 600 s. Additionally, the CF/PPS composites exhibit a lower D_m_ value than CFRP and PA610. Figure 10 shows images of the CF/PPS composite samples before and after the smoke density tests, demonstrating that no delamination, expansion, shrinkage, or melting occurs.

#### 3.4.3. Toxicity

Toxicity is one of the main causes of death in cases where fire is involved in the generation of toxic gases [35]. The reference toxicity values for various gases and toxicity test results are listed in Table 4. The gases detected in the combustion of the CF/PPS composites included carbon dioxide (CO_2_) and carbon monoxide (CO). The composites tended to decompose thermo-oxidatively in the presence of oxygen and at high temperatures, which contributed to the release of CO and CO_2_. However, hydrogen chloride (HCl), hydrogen bromide (HBr), hydrogen cyanide (HCN), hydrogen fluoride (HF), sulfur dioxide (SO_2_), and nitrogen oxides (NOx) were not detected. The R value was obtained to elucidate the final toxicity of the samples. This value is a dimensionless index that provides information on the overall toxicity of all the combustion gases analyzed. For the CF/PPS composites, R was 0.06. A value of R above 1 corresponds to the risk of a person dying after 30 min of exposure to a harmful gas [39,40]. The samples used in this study showed R values of 1 or less; therefore, death by suffocation due to harmful gases would not occur in the event of a fire.

## 4. Conclusions

In this study, we investigated the manufacturing of thermoplastic CF/PPS composites using induction-heating molding.

(1) The void content of the CF/PPS composites treated via the induction process at 280 °C was lower than 1%, which is an acceptable value for the aerospace industry.
(2) The tensile strengths along 0° and 90°, and ILSS of the CF/PPS composites were 1912.57 ± 262.58 MPa, 42.37 ± 4.52 MPa, and 73.92 ± 0.41 MPa, respectively.
(3) In addition, the CF/PPS composites exhibited a superior fire performance, with a lower PHRR, mass loss rate, and fire load for THR.

These results indicate that the induction-heating molding process is suitable for fabricating internal reinforcement parts such as beams and trusses. The proposed method eliminates the need for an iterative testing process to determine a suitable thermoforming manufacturing method for aircraft parts, thereby facilitating material, time, and cost savings.

## Figures and Tables

**Table 1:** Properties of the CF/PPS UD tape.

Test	Unit	Value
Density	g/cm^3^	1.35
Glass transition temperature (T_g_)	°C	90
Melting temperature (T_m_)	°C	280
Processing temperature (T_p_)	°C	300–330
Tensile strength (0°)	MPa	2020
Tensile strength (90°)	MPa	39

**Table 2:** Reference toxicity values for gases and toxicity test result.

Gas	Reference (g/m^2^)	Results (g/m^2^)
CO_2_	14,000	648.6
CO	280	2
HCL	15	0
HBr	20	0
HCN	11	0
HF	4.9	0
SO_2_	53	0
NO_2_	7.6	0
Toxicity index (R)	0.06	

**Table 3:** Degree of crystallinity of the CF/PPS composites measured by DSC.

Temperature (°C)	Degree of Crystallinity (%)
250	23.1
280	25.3
310	26.2

**Table 4:** Cone calorimeter test results of CF/PPS and other composites (processing temperatures of 280 °C).

Composites	TTI (s)	PHRR (kW/m^2^)	THR (MJ/m^2^)	MARHE (kW/m^2^)	Refs.
CF/PPS	0	2.8 ± 0.1	0.9 ± 0.1	1.1 ± 0.1	This work
CFRP ^a^	78 ± 1	196 ± 9	34.9 ± 0.1	94.6 ± 1.7	[26]
PP ^b^	25 ± 2	1738 ± 78	34 ± 1	-	[27]
PA 610 ^c^	73.5 ± 0.5	743 ± 4	128.1 ± 10.2	337.8 ± 5.2	[28]

^a^ Carbon fiber-reinforced bisphenol F and aniline-based benzoxazine, (50 kW/m^2^, 20 min); ^b^ Isotactic polypropylene, (35 kW/m^2^, 20 min); ^c^ Polyamide 610, (50 kW/m^2^, 20 min).

**Table 5:** Smoke density test results of CF/PPS composites (processing temperatures of 280 °C) and other composites.

Composites	D_s(10)_	D_m_	VOF4	Refs.
CF/PPS	17.7 ± 0.1	17.9 ± 0.1	10.2 ± 0.1	This work
CFRP	418 ± 20	430 ± 37	287 ± 34	[26]
PP	-	-	-	[27]
PA610	364.3 ± 4.2	372.2 ± 5.9	-	[28]

## Data Availability

Not applicable.
